# Supplementary material for: Early screening of autism spectrum disorder using cry features
Source: PLoS One. 2020 Dec 10;15(12):e0241690. doi: 10.1371/journal.pone.0241690 (PMC7728261; doi:10.1371/journal.pone.0241690)
Supplement: Attachment — Submitted filename: Response to Reviewers.docx [file pone.0241690.s001.docx]

Journal Requirements:

When submitting your revision, we need you to address these additional requirements:

1. Please ensure that your manuscript meets PLOS ONE's style requirements, including those for file naming. The PLOS ONE style templates can be found at <http://www.plosone.org/attachments/PLOSOne_formatting_sample_main_body.pdf> and <http://www.plosone.org/attachments/PLOSOne_formatting_sample_title_authors_affiliations.pdf>

2. We suggest you thoroughly copyedit your manuscript for 0ge usage, spelling, and grammar. If you do not know anyone who can help you do this, you may wish to consider employing a professional scientific editing service.  

Whilst you may use any professional scientific editing service of your choice, PLOS has partnered with both American Journal Experts (AJE) and Editage to provide discounted services to PLOS authors. Both organizations have experience helping authors meet PLOS guidelines and can provide language editing, translation, manuscript formatting, and figure formatting to ensure your manuscript meets our submission guidelines. To take advantage of our partnership with AJE, visit the AJE website (http://learn.aje.com/plos/) for a 15% discount off AJE services. To take advantage of our partnership with Editage, visit the Editage website ([www.editage.com](http://www.editage.com)) and enter referral code PLOSEDIT for a 15% discount off Editage services.  If the PLOS editorial team finds any language issues in text that either AJE or Editage has edited, the service provider will re-edit the text for free.

Upon resubmission, please provide the following:

- The name of the colleague or the details of the professional service that edited your manuscript
- A copy of your manuscript showing your changes by either highlighting them or using track changes (uploaded as a *supporting information* file)
- A clean copy of the edited manuscript (uploaded as the new *manuscript* file)

3. We note from your ethics statement that 'The study has been approved by the ethics committee at Shahid Beheshti University of
Medical Sciences and Health Services. All the parents of the subjects were informed about the study and signed an agreement to be included in the study.'

Please include this information in the methods section of the manuscript.

It is included now in lines 112-115.

4. We note that you have stated that you will provide repository information for your data at acceptance. Should your manuscript be accepted for publication, we will hold it until you provide the relevant accession numbers or DOIs necessary to access your data. If you wish to make changes to your Data Availability statement, please describe these changes in your cover letter and we will update your Data Availability statement to reflect the information you provide.

Thanks a lot. We should make a decision on where to upload it and we would change Data Availability statement after the acceptance in the cover letter.

5. Please amend either the abstract on the online submission form (via Edit Submission) or the abstract in the manuscript so that they are identical.

At the time of resubmission the abstract on the online form will be changed to the one in the manuscript.

6. Your ethics statement must appear in the Methods section of your manuscript. If your ethics statement is written in any section besides the Methods, please move it to the Methods section and delete it from any other section. Please also ensure that your ethics statement is included in your manuscript, as the ethics section of your online submission will not be published alongside your manuscript.

It is included now in method section in lines 112-115.

7. Please include captions for your Supporting Information files at the end of your manuscript, and update any in-text citations to match accordingly. Please see our Supporting Information guidelines for more information: <http://journals.plos.org/plosone/s/supporting-information>.

Appendix text in this new version has been moved to the method section.

[Note: HTML markup is below. Please do not edit.]

Reviewers' comments:

Reviewer's Responses to Questions

**Comments to the Author**

1. Is the manuscript technically sound, and do the data support the conclusions?

The manuscript must describe a technically sound piece of scientific research with data that supports the conclusions. Experiments must have been conducted rigorously, with appropriate controls, replication, and sample sizes. The conclusions must be drawn appropriately based on the data presented.

Reviewer #1: No

Reviewer #2: Partly

Reviewer #3: No

2. Has the statistical analysis been performed appropriately and rigorously?

Reviewer #1: Yes

Reviewer #2: I Don't Know

Reviewer #3: No

3. Have the authors made all data underlying the findings in their manuscript fully available?

The [PLOS Data policy](http://www.plosone.org/static/policies.action#sharing) requires authors to make all data underlying the findings described in their manuscript fully available without restriction, with rare exception (please refer to the Data Availability Statement in the manuscript PDF file). The data should be provided as part of the manuscript or its supporting information, or deposited to a public repository. For example, in addition to summary statistics, the data points behind means, medians and variance measures should be available. If there are restrictions on publicly sharing data—e.g. participant privacy or use of data from a third party—those must be specified.

Reviewer #1: No

Reviewer #2: Yes

Reviewer #3: Yes

4. Is the manuscript presented in an intelligible fashion and written in standard English?

PLOS ONE does not copyedit accepted manuscripts, so the language in submitted articles must be clear, correct, and unambiguous. Any typographical or grammatical errors should be corrected at revision, so please note any specific errors here.

Reviewer #1: Yes

Reviewer #2: No

Reviewer #3: No

5. Review Comments to the Author

Please use the space provided to explain your answers to the questions above. You may also include additional comments for the author, including concerns about dual publication, research ethics, or publication ethics. (Please upload your review as an attachment if it exceeds 20,000 characters)

Reviewer #1: The research reported in this article follows a recent trend towards the development of objective and easily implemented biomarkers that can be used to distinguish individuals with vs. without autism and in some cases, identify autism in the first year of life. In this study machine learning and other AI-based tools were applied to children’s cries and were found to have high sensitivity and specificity in distinguishing children with and without autism.
The authors are to be applauded for undertaking a problem of such great public health importance. However, there are several limitations of the work that detracts from its overall impact. These are listed below
a) Autism is a notoriously heterogenous disorder, and as a result, the sample size used in this work is likely not representative of autism generally. If the algorithms developed by this team are as robust as they purport, then they should be applied to publicly available data on a much larger scale.

To the best of our knowledge, there is no public data of crying voices which have the tag for having ASD or not. So, it is not possible to check our approach on other data sets. Due to this limitation we tried to collect data by ourselves. Consequently, there are two points that should be noted:

1. Although autism is a heterogenous disorder and having a large dataset would help to show if the algorithm works on all the samples, our method was trained and tested on a subgroup and worked well. In other words, we showed that it can work on a subgroup of children with autism. This is similar to tip-toe walking which is present in a subgroup of children with autism. Having tip-toe walking in a subgroup of children with autism does not make it a worthless feature for screening children with autism.
2. We have tested the algorithm on 57 more children, as we mentioned in the discussion section, and distinguished two suspicious children. These two were examined by two experts later and they approved the existence of other cognitive disorders, as explained in discussion part, in these two children. In other words, the proposed classifier can do initial screening for children with autism and possibly children with other cognitive disorders.

NOTE: It should be mentioned that the number of this group of participants is the same as before (57) but one boy’s parents did not cooperate during the follow-up and he was removed from the participants. Instead, another boy who was 11 months old and was detected as ASD using our classifier was added to participants. He is indicated in Table 3 as Child1.

b) No mention is made of whether the children in this study had IQ or language in the typical range or whether some had an underlying intellectual disability or speech/language problem, both of which could contribute to atypical cries having nothing to do with autism per se

None of the participants have any evidence of underlying intellectual disability. It is also indicated in inclusion criteria as follows:

“The inclusion criteria of the ASD participants were: a) had been just diagnosed with ASD based on DSM-5 with no other neurodevelopmental, mental, and intellectual disorder, b) there were no other known medical, genetic conditions, or environmental factors, and c) had not taken any treatment or medication more than a month.”

The language range of children with ASD is explained in line 129 as follows:

“The participants’ average language development assessed based on [38-41] was between 6 to 12 months of age at the time of participation.”

For the TD participants, the following has been stated in the ms in lines 136-137

“They had no evidence and official diagnosis of neurological or psychological disorder at the time of recording their voices.”

They were also followed up when they reached over three years of age to see if their developmental criteria were achieved. We used a set of expert-selected questions based on [43] to assess our TD participants through interviews with parents. If a child seemed suspicious they were referred for more assessment. Finally, among TD participants in the training set there were no case with any evidence of neurodevelopmental, genetic, or clinical problems. In the test set population of children under 18 months there were three children with language delay when they were followed up at age 3 and more. To address this in the ms, we added the following sentence in the ms, lines 141-144 as follows:

“Furthermore, all TD participants under 3 years of age were followed up when they passed 3, to make sure the initial TD assignment was correct or still valid. To do so, we used a set of expert-selected questions based on [43] to assess them through interviews with parents.“

c) Similarly, no mention is made of whether any of the children contributing data had an underlying genetic disorder, which again could lead to atypical (and distinct) cries.

It’s a good point which has already been addressed in item b.

d) Without comparing the cries of children with ASD to those without ASD but another disorder, we don’t really know if these findings are specific to autism or to atypical brain development generally.

That’s a very good point and we have been aware of this. The current study suggests that a group of children with autism can be screened from TD ones using this method. Then, they have to be examined by experts to make the final classification. In the future, we would expand the study to investigate if these features are specific to children with ASD and also investigate for probable new features may be more important in presence of data from other disorders.

To clarify this in the paper, we have added the following in the discussion section

“In our study only children with ASD and TD children were tested. Other developmental disorders or health issues were not tested to see how children with those disorders would be classified using the proposed method which can decrease the specificity of 100%. However, this approach is proposed to be used as a screening tool and final diagnosis should be done under experts’ supervision. So, this approach can be applicable as a general screener of autism spectrum disorder.”

e) Little attention is seemingly paid to the context for children’s cries, and this would seem important to understand; thus, were the cries related to distress, to pain, to hunger, to fatigue, etc?

It is addressed in lines 194-5 and are indicated with more detail in column named Reason of cry in Tables 1 and 2. However, if it needs more explanation please let us know.

f) I was a little confused about the gender difference the authors report. My sense is that the training set only involved male cries but the test set involved both males and females? If that is correct, I don’t know why a female training set was not developed.

This is a good point and we would highlight it as a limitation of our study. This is due to the fact that girls are less likely to have autism and consequently, it is harder to collect enough data than male subjects. However, in our future work, we would try to collect more female data to be able to train a system to accurately screen females.

It should be noted that we tested the trained system on the female subjects and showed that it can screen female children with lower accuracy. In other words, it seems that gender matters and it should be considered in the training of the system.

To clarify this, we had modified the following sentences in lines 118 to 123 in the manuscript.

“Since we expected to have different vocalization characteristics for boys and girls, the training set was assembled of only boys, including10 TD, and 10 ASD. In other words, we wanted to eliminate the gender effects on the feature extraction and model training. Unfortunately, due to the lower number of girls with ASD in the real world, not enough data for girls with ASD could be collected. Nonetheless, the model was also tested on the girls to see how it would generalize even on girls.”

Additionally, in conclusion we added the following sentences in lines 435 to 445 in the manuscript.

“Due to the fact that girls are less likely to have autism and consequently, it is harder to collect enough data from girls than boys, the number of girls with ASD was not enough to train a separate classifier for this gender. It should be noted that we tested the trained system on 7 girls with ASD and 7 TD girls. It was shown that the trained classifier can screen girls with 7% lower accuracy than boys of the test set. In other words, it seems that gender matters and it should be considered in the training of the system. In testing the data from participants under 18 months, one TD girl was classified as ASD which was not the case for any TD children of the male counterparts. This result also confirms the mentioned note about the gender effect. However, in our future work, we would try to collect more data of girls to be able to train a system to accurately screen girls. In the future, we would also try to train a single classifier for boys and girls to determine whether it can be used for both of them.”

g) Finally, in several places in the text the authors appear to conflate screening for autism with early identification of autism; these are two very different problems.

Thanks a lot for the point. All “identification of autism” is changed to “screening autism”.

Reviewer #2: Thank you for the opportunity to review the manuscript “Early screening of autism spectrum disorder using cry features”. This study developed a new method for distinguishing children with autism from typically developing children, based on two different features of their cries. The method involved training a model on cry features from one set of children, and then testing the model on a new set of cries from different children. The results (e.g., high sensitivity and specificity) are impressive, and the implications are intriguing. However, there are several details that are unclear and I have some concerns that might limit the applicability of this research, unless the authors can address them. These major concerns are described below, followed by some minor suggestions that will improve the readability of the manuscript.

**Major concerns:**

1. After reading the paper, I still do not have a clear understanding of the cry features that were useful for screening for ASD. As I understood it, MFCC and SONE are both related to power/amplitude/loudness of the cry, and the features that you used are based on the variance in MFCC and SONE from one temporal window to the next. So is this something that would be perceptible to a human ear? Is it a quality that can be described in terms of vocal quality (e.g. “shrillness”, “hoarseness”, “raspiness”)? Is the quality specific to cries, or would you also expect to see similar characteristics in the vocal quality of children who are talking rather than crying? You do not need to answer each of these specific questions, but if it is possible to describe the results or implications in layman’s terms, I think many readers would appreciate it. On the other hand, if it is something that only a computer can detect with no discernable difference to a human, then it might be informative to say that (e.g., a single sentence in the discussion).

As the reviewer correctly mentioned, both MFCC and SONE are related to power spectrum of speech signal. SONE measures loudness in specific Bark band. While, MFCC is the inverse DFT of log-spectrum in Mel scale. It is believed that MFCC is related to the timbre of the voice. Therefore, F0, MFCC and SONE can be interpreted to be related to pitch, timbre and loudness of a tone.

If a signal is constant or changed linearly over time, the variance of temporal difference is zero. Therefore, the variance of temporal difference can be seen as the amount of ambiguity or unpredictability of a sound.

On the other hand, we tested the voices with one of our experts who was experienced in diagnosing children with autism and therapy for about 10 years whose ear was sensitive to ASD children cry differences. To compare the proposed approach with human expertise in recognizing the crying voice of children with ASD, the cry voices were classified by an autism expert. It should be explained that she listened to the whole raw data voice. It was impossible for her to detect difference between the voices using the cleaned samples. The specificity, sensitivity, and accuracy of her recognition were 83.87%, 80.64%, and 83.87% respectively on the whole dataset (including the training set for our system). This shows that, her sensitivity is very close to us. She reported that there was something which makes the cry of children with ASD more unpredictable and had more variety than TD children.

To address the reviewer’s comment, we added the following to lines 452 to 462 in the text:

“The two discriminative features found in this study were a coefficient of MFCC and a SONE coefficient. MFCC and SONE are related to the power spectrum of a speech signal. SONE measures loudness in specific Bark bands [50]. On the other hand, MFCC, which is the inverse DFT of log-spectrum in the Mel scale, is related to the timbre of the voice [54]. Therefore, MFCC and SONE can be interpreted to be related to the timbre and loudness of a tone. Furthermore, based on the feedback from our experts, there is unpredictability in the crying voice of children with autism which is not true for TD children. Consequently, we used the variance of temporal difference as a feature suitable for screening children with autism. This is due to the fact that if a signal is constant or changed linearly over time, the variance of temporal difference is zero. Therefore, the variance of temporal difference can be seen as the amount of ambiguity or unpredictability of a sound.”

2. If children were crying for different reasons, wouldn’t that affect cry quality? For example, do you know if a hunger cry has the same qualities as a cry of pain? Unless you can verify that children with ASD produce the characteristic cry qualities regardless of the reason for crying, then it would be important to know which type of cry would be most accurate when actually screening a child.

This is a very good point and we considered this in our study. In the application that we developed to collect cry voices, we ask each user to indicate the reason behind the cry. Also, in our study, we found out that the cries initiated based on pain are not suitable for differentiating children with autism from TD ones and this point is indicated in lines 194-5 of manuscript. Thus, these cry voices were removed from the study. The list of reasons behind the used cry voices are given in Tables 1 and 2.

3. If children in the training sample produced different numbers of cries, wouldn’t the children with more cry instances be weighted more heavily in the model than children with fewer cry instances?

To avoid this problem, we used upsampling to make avoid giving more weight to one subject over the others. This has been mentioned previously in appendix as follows:” Before applying the algorithm, we balance the number of instances of the two groups by upsampling.” It should be noted that in new version of the ms the text of appendix has been moved to the Method section due to a reviewer’s advice. So in this new version of the ms this explanation is in lines 315-316 and 323-324.

4. The children in the TD group were not assessed to verify that they were all truly typically developing. So this group could have included children with ASD that had not yet been diagnosed, or children with some other non-ASD disorder. This is not a fatal flaw, but it should be mentioned as a possible limitation. Specifically, it limits your ability to say whether this method distinguishes children with ASD from all other non-ASD children (i.e., including TD children and children with other disorders) or if it simply distinguishes all non-TD children from TD children.

This is a very good point. Although we did not run sophisticated tests to assess TD children, we used a set of expert-selected questions based on developmental milestones indicated in a reference [43], to assess our TD subjects. If a child seemed suspicious, he/she were referred for more assessment. This is also done for the children who were under 3 years of old at the time of voice recording, when they reached to 3 or more years of old. To clarify this for the readers, it has been added in the participants subsection in lines 136-137 and 141-144 as follows:

“They had no evidence and official diagnosis of neurological or psychological disorder at the time of recording their voices.”

“Furthermore, all TD participants under 3 years of age were followed up when they passed 3, to make sure the initial TD assignment was correct or still valid. To do so, we used a set of expert-selected questions based on [43] to assess them through interviews with parents.”

Finally, in the set of children under 18 months there were five children who were not normal when they were followed up at the age of 3 and more. A brief report for the participants under 18 months for whom there were some concerns about their normal developmental progress or any disorder after the age of 3 included in Table 3.

5. Related to the last point, the children were not followed up later, so you do not know if some of the TD children were diagnosed with ASD (or some other disorder) at a later age. If any of the TD children were diagnosed at a later age, this would decrease the sensitivity. In general, the method seems to be only as sensitive as the person who was providing the evaluations (rather than more sensitive, so it can detect children with ASD *before* they have obvious symptoms).

That is a very good point. It may be possible that a child without obvious ASD symptoms have specific cry features. However, our goal in this study was to determine if a child has ASD or not at the time of test using his/her cry voices. We may address this issue in our future work. It should be mentioned that all TD children whose voices were recorded when they were under 3 years old, were followed up and in this new version of the manuscript the following up results are included as mentioned in previous response.

Among all children there was one child with no evidence of having ASD at the time of voice recording (at the age of one year) and he was diagnosed with ASD when he was about 2 years old. His initial voice was not detected by our trained classifier and this should be discussed. To discuss it two following paragraphs in the discussion section are addressed as follows:

“The trained classifier was also tested on 57 participants between 10 to 18 months of age. The classifier screened two boys from the rest, i.e. Child1 and Child2 (Table 3). Child1 showed evidences of genetic disease and diagnosed with developmental delay and Child2 received UNDD classification by our experts. This suggests that a) the system can be used for children under 2 years of age, and b) it may be able to distinguish other neurodevelopmental disorders. On the other hand, there were 5 boys, i.e. Child3 to Child7 (Table 3), who had no evidence of mental or developmental disorders at the time of their recording. At the same time, our approach did not distinguish them as children with ASD either. However, when they got older than 3 years, they showed symptoms of neurodevelopmental disorders. From these children, Child3 and Child4’s voices, collected after receiving the diagnosis, were classified as children with ASD using our approach. Unfortunately, Child5, Child6, and Child7 did not cooperate to be evaluated by an expert to validate the results of our expert-selected questionnaire. Furthermore, they did not cooperate to send us their children’s recent cry voices.

The result of studying these 57 children under age of 18 months may suggest that: a) there could be symptoms in the crying voices of children with neurodevelopmental disorders under 18 months (Child1 and Child2), b) the approach may not be able to screen a participant with neurodevelopmental disorders under the age of 18 months with the possibility of: 1) the participant was among those children with neurodevelopmental disorder who do not have our proposed specific features in their crying voices, 2) the participant’s recorded cry samples did not include our specific features, and/or 3) Neurodevelopmental disorders and their features had not been developed in the child at the time of initial recording. The reason behind not classifying Child3 and Child4, as children with ASD under the age of 18, could be b.2 or b.3. To clearly determine the reason behind this phenomena, further investigation is needed. ”

NOTE: It should be mentioned that the number of this group of participants is the same as before (57) but one boy’s parents did not cooperate during the follow-up and he was removed from the participants. Instead, another boy who was 11 months old and was detected as ASD using our classifier was added to participants. He is indicated in Table 3 as Child1.

6. The current sensitivity & specificity are impressive, but this may be because you included only TD and ASD groups. In real-world settings (e.g., pediatrician offices), this method would need to be able to classify other groups of children too, such as those with health issues (e.g., asthma) or other developmental disorders (e.g., apraxia). It is unclear how these other groups (especially groups with atypical speech, like apraxia of speech or a stutter) would be classified by this method. If these children were classified as “not TD” that would drastically reduce the specificity of this method as an ASD screener, but might make it useful as a general screener that would then lead to a more comprehensive evaluation.

This is a very good point. However, our goal was to show that a class of children with autism can be screened using this method. Further study is needed to examine the approach on children with other disorders. Our goal is to extend the work on this in the future. To clarify this for readers, we have added the following in the discussion section, lines 471-475:

“In our study only children with ASD and TD children were tested. Other developmental disorders or health issues were not tested to see how children with those disorders would be classified using the proposed method which can decrease the specificity of 100%. However, this approach is proposed to be used as a screening tool and final diagnosis should be done under experts’ supervision. So, this approach can be applied as a general screener of autism spectrum disorder.”

7. The goal is to diagnose children earlier than is currently possible, but the model is trained on children that are approximately 3 years of age (which is around the current average age of diagnosis). It would be important to know how accurate this method would be if used at 12 months, 18 months, or 24 months. You have begun to address this by testing the model on 61 children between 10 and 18 months of age, but the real test of sensitivity would require you to evaluate these 61 children after 3 years of age, so that you could say that the one child that was detected was truly the only one that was not TD.

We are thankful for the very constructive suggestions. We have followed up all the children who were under 3 years old at the age of 3 or more. The results for those who had any problem are given in Table 3. The results of the classifier detection on them is also reported in the results section in Table 8.

**Minor suggestions:**

Pg. 1 & Pg. 8: The abstract on page 1 is completely different from the abstract on page 8 of the PDF.

Thanks a lot for the point. It is sgoing to be fixed in the new submission.

Line 21: “correspondign” is misspelled

Line 27: “feamale” is misspelled

Line 34: “ignited” is metaphorical. A literal word might be better, such as “inspired” or “instigated”

Line 35: instead of “many researches”, try “much research”

Line 38: I would change “about 3 years” to “over 3 years”, because the reported average age of 3.1 years was just for the most severe cases. Less severe cases of ASD (e.g., PDD-NOS and Asperger’s) were diagnosed at much later ages (3.9 and 7.2 years, respectively).

Line 53: “features differences” could be changed to “feature differences”

Thanks for the points. They are modified after your mentions.

Lines 58-59 (also 73-74): This method is unfamiliar to me. I feel confident that I understand what you mean, but it might be useful to include a citation.

That’s a good idea and it is included in line 75 of the new version of the manuscript now.

Lines 94-97: I find this section to be unnecessary. All of this is redundant with the sub-headings below. This might be helpful if the method was very long or complicated, but I think yours is fairly straightforward.

You are right. Those redundant lines are omitted.

Line 103: “set” should be plural, “sets”

Thanks. It has been modified.

Line 104: instead of “only a model was trained for screening male subjects”, try “the model was only trained for screening male subjects”

Thanks. It has been modified.

Line 112: “psychologist” should be plural, “psychologists”

Thanks. It has been modified.

Line 113: “was” should be added, to make it “diagnosis of ASD was established”

Thanks. It has been modified.

Lines 123-132: some of the column headers are self-explanatory and do not need to be described in the text (e.g., subject IDs, ages). The only time you need to explain further is when there is information that is not explained in the table, such as the reason for two recording device types, or the 4 participants without GARS scores.

Thanks for the point. It seems the ID and Age columns do not need further explanations and we omit them.

Line 129: Does “GRAS grades” mean “GARS scores”?

Yes for sure. We made all of its usage the same in the paper after your mention.

Line 149: “balancing on” should be “balancing of”

Thanks. It is changed.

Line 162: “there were asked” should be “they were asked”

Thanks. It has been modified.

Line 174: Why were the uvular/guttural parts of the cries removed? Is there evidence that these parts are not informative? If so, you could cite that evidence. Otherwise, you could add “because we believed [whatever the reason was]”

For sure because we believed from data analysis we did. It is explained in the ms now as follows:

“The reason is that we believe these parts distort the feature values of the steady parts of a voice.”

Line 177: what does it mean “to be comprehended by our audition?”

The sentence has been removed to fully explain the process and address the reviewers’ feedback. The further explanation is given below. Since the initial idea came from our human experts who could recognize cry voices of a set of children with ASD, we decided to include segments that we could recognize them as a cry voice ourselves. Thus, we performed an experiment on a few people and determined that cry instances around 300 milliseconds could be recognized as cry voices. Furthermore, since the basic voice features are extracted from 20 milliseconds frames, to generate statistical features between several 20 milliseconds frames in a continues segment provided by preprocessing phase, we set to have minimum of 15 frames, i.e. 300 milliseconds, in each cry voice. In other words, the basic features are extracted from equal length cry frames while the statistical features are calculated from minimum of 15 frames.’

We add this explanation instead in lines 205-209 and the paragraph is re-written in this way:

“Finally, since the basic voice features were extracted from 20 milliseconds frames, to generate statistical features of the basic features, the minimum length of the cry segments were set to 15 frames, i.e. 300 milliseconds. Thus, any cry samples below 300 milliseconds were eliminated from the study. In this study, the final prepared samples were between 320 milliseconds to 3 seconds.”

Line 229: “till” should be “until”

Thanks. It has been modified.

Line 230: “is appeared” should just be “appeared”

Thanks. It has been modified.

Line 240: In my opinion, this whole “Feature extraction” section would have been helpful if it was presented before the section about classifying based on features.

If you mean the section of proposing the method, it is now moved before that section.

Line 255-256: I do not understand why you modified the spectral flatness features. If this is common practice, you could either just say that or provide a citation. If it was your own decision, you could say why you decided that it was necessary.

“We modified the spectral flatness features by including the range of 125-250 Hz beside the 250-500 Hz range. This range was added to cover a wider frequency range than the normal children frequency range, which showed to be necessary in the process of feature extraction and selection.”

We also add this explanation in ms in lines 226-229.

Line 270: “omitted by” should be “omitted for”

Line 271: I think this should have been said earlier, such as after line 249. While I was reading lines 253-255, I had been wondering how long a frame was.
Thanks a lot for your good point. It is now placed there which is lines 206 and 220 in the new version of ms.

Line 279: “details)” has a parenthesis attached

Thanks. The whole word is omitted now as it was already redundant.

Lines 280-281 (and line 284, as well as lines 300-301): These are technical terms that I am not familiar with. Would it be useful to provide a citation?

In this sentence “These features are Variance of Frame-wise Temporal Derivative (VFTD) of the 7th MFCC coefficient and VFTD of the 6th SONE coefficient (Fig 3)”, there is “Temporal Derivative” which has now references added that used temporal derivative for some speech feature in it and the reference is added in the first occurrence of the word in line 224 in the sentence “They also used the mean and variance of frame-wise temporal derivative [49, 50] of the basic features.”

SONE and MFCC have references as well in lines 348 And 217 respectively.

In the sentence “On the other hand, the best-resulting classifier for the WSI approach is Radial Basis Function-Support Vector Machine (RBF-SVM)” a reference at the end of the sentence is added now in line 369.

For the sentence “To classify each participant using the WSI classifier, the Majority Pooling (MP) and the Best-chance threshold Pooling (BP) approaches are used. BP is a threshold-based pooling with the threshold giving the best accuracy on the test set for male participants.” The explanation of “majority pooling” is in lines 318-319 and “threshold-base pooling” is explained in lines 319-321.

Line 336: what is meant by “the only method”?

It is not clear by the text. Thanks for letting us know it. We mean the only method’s introduced features in the literature which used cry features.

The sentence in the ms is modified to:

“We compared the results of our proposed method with that of the only method available in the literature which was trained using only cry features [36] based on our data(Table 7).”

Line 353: “was used to train” should be “were used to train”

Thanks. It has been modified.

Line 358: “subject” should be plural, “subjects”

Thanks. It has been modified to “participants” generally.

Line 368: I am not certain, but I think “claimed” should be “claims”

Thanks, you are right.

Lines 372-373: I think this is good, if it is intended to screen for atypical development in general. But if this method is meant to be specific for ASD screening, then this would result in an overall decrease in specificity.

Thanks for the very important point. Since we have only aimed at children with autism, we cannot claim for any other neurodevelopmental disorder. To address this, we have put the following sentences in the discussion section.

“In our study only children with ASD and TD children were tested. Other developmental disorders or health issues were not tested to see how children with those disorders would be classified using the proposed method which can decrease the specificity of 100%. However, this approach is proposed to be used as a screening tool and final diagnosis should be done under experts’ supervision. So, this approach can be applied as a general screener of autism spectrum disorder.”

Lines 480 (and elsewhere in the Appendix): you used some technical terms but not citations are provided.

Thanks for the point. A reference to cross validation method which include K-fold methods too, is added to the ms at first occurrence in line 75.

Reviewer #3: This manuscript addresses an important question: identifying children with ASD manifestations using biological information, specifically, auditory signals from crying. One compelling result is heightened variability in ASD cries relative to TD children. The interpretation (though now on P. 24 in the Appendix, and needs to be more fully elaborated and presented sooner in the ms) is that the findings are not due to cry per se, but due to the specific statistical features of the cry instances that capture heighted variability of cries. The authors propose that because the increased variability may be an enduring, fundamental feature of ASD, for this reason the pattern holds constant across age. I agree that heightened variability in biological signals is a clear and an emerging trend in ASD and I think that this manuscript does add important new information for our understanding of ASD in toddlers.

There are several major technical/scientific and conceptual concerns that should be addressed though. One example (there are many, see below) of a technical concern is a lack of uniform processing strategy for cry instances, resulting in a huge range of cry durations (1/3 to 3 seconds). In addition, this manuscript requires substantial re-drafting to incorporate more recent references and rationale for certain points made, and other places require additional justification and elaboration. The text is somewhat sloppy and not well-prepared (however, this issue could be related to the language use concern noted below). Limitations should be articulated.

Please have a colleague or a student who is a native British English or American English speaker carefully review and edit the entire manuscript, including captions and appendices. If this is not possible, please use an English-language editing fee-based service. The language use issues (e.g. awkward language and word use; grammar) are significant, numerous, and should be addressed adequately prior to re-submission.

Despite these points, I think that the work has important strengths. I also think that given the importance of question on early ASD detection, the authors may be given an opportunity to revise the ms, provided that they carefully consider and address all points raised (for any points not addressed, please provide suitable rebuttals).


abstract

-there are currently 2 versions of the Abstract text – one provided along via the submission system and a second version included as part of the manuscript file.
1) Please review language usage in both and 2) please choose one version (not clear which is the final version?)

Thanks for pointing this out. It has been solved and the new version would be used in both cases.


“The approach has been tested on a dataset including 14 male and 7 female children with ASD and 14 male and 7 female TD children, between 18 to 53 months of age.”
-but the algorithm was developed with male subjects data only? Why? What is the result when you test on male-only subset?

As it is indicated in lines 120-123, it was aimed to eliminate the gender effect on the features. Thus, we investigated male and female samples separately. Unfortunately, due to the fact that the number of girls with autism is much less than males with autism, we did not have enough female samples to train a model for females. We are currently in the process of collecting more data female participants to train a model for female children. It was noted in the manuscript that the model for male children was able to distinguish the female children with autism from TD ones at a lower accuracy.

This explanations are included in the participants subsection and the discussion section as follows:

“Since we expected to have different vocalization characteristics for boys and girls, the training set was assembled of only boys, including10 TD, and 10 ASD. In other words, we wanted to eliminate the gender effects on the feature extraction and model training. Unfortunately, due to the lower number of girls with ASD in the real world, not enough data for girls with ASD could be collected. Nonetheless, the model was also tested on the girls to see how it would generalize even on girls.”

“Due to the fact that girls are less likely to have autism and consequently, it is harder to collect enough data from girls than boys, the number of girls with ASD were not enough to train a separate classifier for this gender. It should be noted that we tested the trained system on 7 girls with ASD and 7 TD girls. It was shown that the trained classifier can screen girls with 7% lower accuracy than boys of the test set. In other words, it seems that gender matters and it should be considered in the training of the system. In testing the data from participants under 18 months, one TD girl was classified as ASD which was not the case for any TD children of male counterparts. This result also confirms the mentioned note about the gender effect. However, in our future work, we would try to collect more data of girls to be able to train a system to accurately screen girls. In the future, we would also try to train a single classifier for boys and girls to determine whether it can be used for the both of them. ”

Introduction

“On the other hand, it is shown that fMRI [10] or EEG [11] can give discriminative features helping to diagnose ASD at earlier ages.”
This statement is not accurate, as ref [10] predicted future ASD diagnoses made by traditional means (ADOS, ADI-R), by using rs-fMRI data from 6 mo. The diagnoses themselves are still performed using observational instruments. This approach is not the same as one to be used for “screening” for ASD symptoms in the absence of a diagnosis and also it is not the same as one to be used for using biobehavioral information in lieu of conventional diagnoses. Please be careful in your writing and distinguish (1) work reporting results that predict future ASD diagnoses/cases, or (2) work that is able to detect concurrent cases of ASD (i.e. diagnoses are made at the same time as classification), or (3) work that aims to establish biological features of ASD that can be themselves be used for ASD diagnoses in lieu of traditional instruments.
Here it may be helpful to add ref Denisova & Zhao, 2017 who used movement data from rs-fMRI from 1 mo to predict future atypical developmental trajectories in general (this report provides the earliest age of detection using rs-fMRI data in an unconventional way).

Thanks for pointing this out. We have removed the term diagnosed and replaced it with the following:

“On the other hand, Emerson et. al showed that fMRI [16] can be used to predict the diagnosis of autism at 2 years of age of high-risk 6-month-old infants. Denisova and Zhao [17] used movement data from rs-fMRI from 1-2 month-old infants to predict future atypical developmental trajectories as biological features. Furthermore, Bosl, Tager-Flusberg, and Nelson [18] suggested that useful biomarkers can be extracted from EEG signals for early detection of autism.”

Please also add more recent references from various groups who report ASD classification/ screening using items from the ADOS that is more recent: Kuepper et al., 2020; Abbas et al., 2020. Also add more references for younger ages since that is your target population.

Thanks again for the given references, we have added them to the paper in lines 45-50 as follows:

“To reduce the dependency on the human expertise needed in using such questionnaires [8], several studies proposed machine learning methods to classify children with ASD [13, 14] using questionnaires. Their goal is to make the process automatic and/or find an optimum subset of questions or features. For instance, Abbas et al. [15] proposed a multi-modular assessment system combined of three modules, a parent questionnaire, a clinician questionnaire, and a video assessment module.”

Unfortunately, we could not find studies in this area for children under 18 months.

“Furthermore, approaches which involves methods such as fMRI or EEG, are hard to be used on children, especially children with autism.”
This statement requires support from the literature – what makes them “hard” or unsuitable? (excessive head movements, etc. Please provide specific reasons and supporting references for each point made) (e.g. Denisova 2019).

To support the statement, two references which indicates there are considerations needed in collecting data with children and children with autism using fMRI and EEG are added to the manuscript after the sentence you mentioned.

It is modified as follows:

“Finally, approaches which involve methods such as fMRI or EEG, are hard to be used on children, especially on children with autism who may have trouble following instructions appropriately [22], have atypical behaviors [23], or have excessive head movements [24, 25].”

Oller et al. 2010 paper should be discussed in the Introduction.

It is mentioned in lines 75-78 as follow. Are you suggesting to give more explanation of the work?

” Oller et al. [29] proposed another vocalization-based classification method in which they included age and excluded crying. They applied the method on 106 TD children and 77 children with ASD between 16 to 48 months and reached 86% accuracy.”

P. 2 Introduction
“To the best of our knowledge, there is only one work that proposed a method for identifying ASD children using only cry [24]. They used a dataset of 5 children with ASD and 4 TD children older than two years of age. They extracted 187 sound features from which 55 features were selected, using forward selection.”

This reference #24 seems to be work from the same group as authors of the current manuscript. Please refer to this reference appropriately (“We” instead of “They”, or “Work from our group has shown that …”)

The reference number is now 36. It has been fixed as follows:

“To the best of our knowledge, our own group’s preliminary study [36] was the only research that used cry voices for the screening of children with ASD. We used a dataset of 5 children with ASD and 4 TD children older than two years of age. The accuracy of the proposed method is 96.17% using k-fold cross validation without considering subject-wise hold out, which is a shortcoming of this study. In other words, it has been overfitted to the available data and may fail to correctly classify new samples. So, a thorough examination using an unseen test set on cry features is necessary to evaluate the results.”

Is there an overlap of children from study described in Reference #24 with the current work? Please clarify.

No, the data are different. It has been fixed in the paper in line 90-92 as follows:

“. It should be noted that the data from our previous study [36] could not be used in this study due to the differences in data collection procedures.”

P. 3 Introduction
“It should be mentioned that the extracted features are age invariant and have been used to screen children with ASD between 18 to 53 months of age.”

This point about the current method’s age-invariance is emphasized repeatedly as being a strength of the proposed approach, but as written, it is unclear why or even if this might be the case.
It may be problematic in terms of specific behaviors not present at all ages. For an approach to be scientifically valid, it must have ecological validity with respect to natural human developmental behaviors.
For instance, you may encounter fewer crying instances at older ages, even if the older child is undergoing therapy (these children may adapt a different response instead of crying, and throw temper tantrums and present loud yelling and vocalizations).

To address this important note by the reviewer, we have changed the paper so the age invariant note has been removed. For instance, the sentence in the introduction has been changed to:

“It should be mentioned that the final selected features are common among our children with ASD between 18 to 53 months of age.”

In the conclusion, we have also changed the sentence in line 447-8:

“The features found in this study, were applicable in a range of ages of our participants from 18 to 53 months.”

The link to the fact that the statistical feature used (e.g. capturing heightened variability) is or could be feature of ASD that may be age-invariant is not really articulated, until the last page in the Appendix.

We have addressed this in the introduction referring to lines 104-107 to better justify the approach. The added sentence is:

“It should be mentioned that the final selected features, in this study, are common among our set of children with ASD between 18 to 53 months of age. These selected features support experiential knowledge of our experts stating that the variations in the cry of children with ASD are more than TD children.”

It is also addressed in discussion section, lines 456-466, as follows:

“Furthermore, based on the feedback from our experts, there is unpredictability in the crying voice of children with autism which is not true for TD children. Consequently, we used the variance of temporal difference as a feature suitable for screening children with autism. This is due to the fact that if a signal is constant or changed linearly over time, the variance of temporal difference is zero. Therefore, the variance of temporal difference can be seen as the amount of ambiguity or unpredictability of a sound. On the other hand, the heightened variability in the two features, found in this study, for children with ASD is significant due to the reports from other studies [17, 55] which shows increased biological signals variability in children with ASD and infants at high risk for autism in comparison to TD children. These features are statistical features of the cry instances that hold constant, at least, across an age range studied in this research.”

Crying (and voice/speech) is a motor act. Research from various groups has now established that infants, children, adults with ASD have problems in the motor domain and in coordination of motor domain with other modalities. Please cite references to this highly relevant work and please discuss in the Discussion.

That is very interesting point and we had this in mind. However, we could not find a reference to clearly link these two together. Thus, we have added the following into the discussion section as follows:

“It is shown that crying consists of intricate motor activities [56]. On the other hand, it is shown that children with ASD have problems in the motor domain and in coordination of their motor capabilities with other modalities [57]. Consequently, it is possible that the extracted features in the crying voices of children with ASD come from this deficiency/problem in the motor domain which needs further investigations.”

P. 4 Subjects

Please review ascertainment of and description of ASD diagnoses. Please re-write this section in a more succinct, formal prose.

Thanks for pointing this out. It has been fixed as follows:

“The inclusion criteria of the ASD participants were: a) had been just diagnosed with ASD based on DSM-5 with no other neurodevelopmental, mental, and intellectual disorder, b) there were no other known medical, genetic conditions, or environmental factors, and c) had not taken any treatment or medication, or had taken treatment less than a month.”

“The inclusion criteria of the ASD subjects were those who had been just diagnosed as autistic, based on DSM V . . .”
This statement is not correct.
-DSM-5 provides for diagnoses of Autism Spectrum Disorder only. “Autism” is not one of the diagnoses in DSM-5.
-also, please note: the correct name for the fifth DSM version is DSM-5, not “DSMV”.

Thanks a lot for the points. They are modified after your mentions.

-Please provide justification why ADOS (Toddler Module or Module 1), and/or ADI-R was not given. Is there a valid/official translation of the ADOS in Farsi that can be administered in Iran? Is there a research version available that you can request permission to translate from WPS, the publisher? If not, this information should be incorporated, as we clearly need version of ADOS (and/or ADI-R) for Islamic countries. However, as this is a limitation of the work, you should to provide a more thorough explanation of why the assessments are missing from your study and what are the plans to address this important issue in the future.

-I just checked for available official/published translations on the ADOS website and I did not see a Farsi/Persian version.
<https://www.wpspublish.com/published-translations>

-Right now from the text as written it is not clear why these instruments were not given to children in this study.
“For full descriptions of these procedures, see our previous work [27].”
Please do not refer the readers to these key details elsewhere. You may write this section in a more succinct manner but crucial information and justification must be given here in this ms, especially as there are no strict word limitations in PLOS One.

As you have also checked, the official version of ADOS is not available for Farsi. There are different approaches taken to evaluate participants in Iran. In our study, the assessment process was as follows which has been added in the text.

“The autism diagnosis procedure started with the Gilliam Autism Rating Scale-Second Edition (GARS-2) questionnaire [42] which was answered by the parents. Then the parents were interviewed, based on DSM-5, while the participants were evaluated and observed by two Ph.D. degree child clinical psychologists. In addition, the diagnosis of ASD was separately confirmed by at least a child psychiatrist in a different setting.“

p. 6 Table 1.
Typically Developed –> “Typically Developing”. The name of this normal control children’s cohort should be revised to state “Typically Developing”.

Thanks for correcting. It is fixed now.

All in the ms has been changed to typically developing.

“Subjects” – ‘participants’, ‘participating children’.


P. 7, end of the Subject section
This section is not well-prepared and some information (on the devices) is explained in the following section. Please re-draft and re-organize this material in these adjacent sections.

“Furthermore, the number of subjects with high-quality recordings was 6 ASD and 4 TD.”
This statement indicates that the main portion of the data was acquired with typical cell phones. Please re-run the main analysis, including only participants’ data obtained with cell phones.

The sentence that you mentioned is about the training set only and the paragraph is explaining the training set data. As it is explained in the sentence “the number of subjects with high-quality recordings was 6 ASD and 4 TD.” Which means 4 remained ASD and 6 remained TD children’s voices were recorded using cell phones. As it was discussed before that “two groups were as balanced as possible with respect to the age and recording device”.

To better explain the study, we re-wrote the sentence as follows:

“Two groups of 10 TD and 10 ASD children were selected for training the classifiers such that two groups were as balanced as possible with respect to the age and the recording device. Thus, each child in the TD group had a corresponding child in the ASD group with about the same age.”

P. 7, 8 Data collection and preprocessing

Need to provide more details about how the preprocessing step was implemented.

This part is added to the ms:

“To explain more, the parts of cry voices which were accompanying screaming, saying words/other vocalizations, or were occurred with closed/non-empty mouth were eliminated. All segmentations and eliminations were done manually using Sound Forge Pro 11.0. From the selected cries, the beginning and the end, which contain voice rises and fades, were removed to keep only the steady parts of the cries. It prevents having too much variation in the voice which can lead to unsuitable statistics.”

Raw data handling: need to clearly state which stages were automatic and which were manual.

In this study, the preprocessing is done completely manually using Sound Forge Pro 11.0 software.

Data samples: What are they being equated upon? Equal duration?

“The reason for using various devices was to have a device independent model.”
This statement holds if and only if you have already validated your algorithm on some standard data acquisition device (i.e. a high-quality recording device or a cell-phone with high quality audio recording). You need to be confident that the data are acquired in a robust and consistent way. After this step is demonstrated and findings are robust, then you may include a variety of recording devices. For this reason, it would be important to run an analysis using cell-phone data only, as cell-phone acquisitions represent the majority of your data.

It should be mentioned that we used both devices and their data was used together for both training and testing. In other words, we did not limit ourselves to one device to avoid biasing the approach to a specific device. However, as you correctly mentioned in the following paragraph, we should not claim that it is device invariant. Thus, we have fixed the write up in lines 187-188 to address this issue as follows:

“The reason for using various devices was to have a device independent model.” Is modified to “The reason for using various devices was to avoid biasing the approach to a specific device”

It should be noted that the cellphone data is collected using various cell phones as parents used their own cell phones.

P. 8.
“In this study, the final samples were between 320 milliseconds to 3 seconds.”
It is not acceptable to have such a wide range of data length (1/3 s to a full 3 s). Have you run an analysis that rules out data length as a contributing factor? What about the number of instances per each subject/participant? You need to have a similar number of instances across subjects.

This is a good point. To avoid the difference in the number of instances, we used up-sampling to have the same number of instances for each participant. On the other hand, since the basic features are extracted from 20 millisecond frames, the samples length is not a contributing factor.

Please provide clear justification for all analytic choices made. Please select one or more criteria, such as equal length of a cry instance, or the number of discrete crying instances, etc.

Since the initial idea came from our human experts who could recognize cry voices of a set of children with ASD, we decided to include segments that we could recognize them as a cry voice ourselves. Thus, we performed an experiment on a few people and determined that cry instances around 300 milliseconds could be recognized as cry voices. Furthermore, since the basic voice features are extracted from 20 milliseconds frames, to generate statistical features between several 20 milliseconds frames in a continues segment provided by preprocessing phase, we set to have minimum of 15 frames, i.e. 300 milliseconds, in each cry voice. In other words, the basic features are extracted from equal length cry frames while the statistical features are calculated from minimum of 15 frames.

The explanation in the ms is as follows:

“Finally, since the basic voice features were extracted from 20 milliseconds frames, to generate statistical features of the basic features, the minimum length of the cry segments were set to 15 frames, i.e. 300 milliseconds. Thus, any cry samples below 300 milliseconds were eliminated from the study.”

P. 9 Caption of Fig1:
“All instances of a subject have the same color.” – Do you mean to say: all instances *belonging* to a subject/participant?

Thanks for the correction. Yes, for sure. It is modified to “belonging”.


P. 9 “An example of case 1 is tip toe walking in children with ASD, which is common in about 25% of these children who do it most of the times.”
-Please provide a reference from the literature for the tip toe walking example.
-“most of the times” – “most of the time”.

A reference is already referred in the Introduction section for tip toe walking in autism. Another reference to it is provided here again and the correction is made in the ms as follows:

“An example of case 1 is tiptoe walking in children with ASD, which is common in about 25% of these children [37] who do it most of the time.”


P. 13 Please review this technical description for mathematical accuracy:
“Frame-wise temporal derivative means subtracting the value of a frame from the value of the next frame.”
Subtracting value of one frame from the next frame produces a difference of values.

Since the difference is calculated over two next single frames, there was no need to divide the difference by one. Additionally, we should explain that the word “derivative” for this kind of difference is usual in speech/voice processing literature. For example in page 167 of the following reference^1^ the term derivative is used:

^1^Rabiner LR, Schafer RW. Introduction to digital speech processing. Foundations and trends in signal processing. 2007;1(1):1-194.

“Finally, to compute the features, each instance was divided into 20 milliseconds frames.”
What is the rationale for 20ms resampling / duration of each frame’s duration? Ideally you need to follow the Nyquist theorem (if you did, this needs be stated) for determining the proper sampling rate for a given phenomena of interest. However, you seem to have a fixed sampling rate of 44.1 kHz for all of the devices. All of this information needs to be reconciled in a technically and mathematically precise fashion and produce correct and concise descriptions for all methods.

In speech or voice processing, 20 to 30 ms frames^2^ are selected as to extract the basic features. In fact, this range is the best range, from experience, to preserve adequate resolution in both the time and the frequency dimensions, simultaneously. Thus, we used the same frame size for feature extraction.

Sampling rate of 44100 Hz is a common sampling rate in many voice/speech applications as it is capable of giving a maximum frequency of 20000 Hz which is the maximum frequency audible by human.

^2^ Paliwal KK, Lyons JG, Wójcicki KK, editors. Preference for 20-40 ms window duration in speech analysis. 2010 4th International Conference on Signal Processing and Communication Systems; 2010: IEEE.

“Also, we added a few other promising features.”
Please create a new Table summarizing fundamental descriptors and rationale for inclusion/exclusion of specific properties in this study.

Due to the good point, we add another table to the ms, in page 14, which summarize the features which are added to or removed from the sets we introduced and referred in the ms. The table is shown as bellow:

|  | Feature | removing/adding | Reason |
| --- | --- | --- | --- |
| Second set | logarithmic energy | Mean statistic is removed | Classification dependency on loudness/power of cry |
| First set | Audio power |  |  |
|  | Total loudness |  |  |
|  | SONE |  |  |
|  | First MFCC coefficient |  |  |
|  | ZCR | The basic feature is removed | The feature’s dependency on environmental noise |
|  | All basic features applicable | mean of frame-wise temporal derivative of the basic features is removed | No meaning for the feature |
|  | MFCC | Coefficients of 14-24 are added | Having higher-order coefficients for vocal cords information as well as vocal tract |
|  | Spectral flatness | A range of 125-250 Hz is added | Covering the low-frequency range of human voice |

P. 20 (2nd page of Discussion and conclusion)

“Finally, the proposed approach was also tested on 57 subjects, 56 TD children and one who diagnosed as Unspecified Neuro Developmental Disorder (UNDD), between10 to 18 months of age. The classifier distinguished the UNDD child from the rest. This suggests that a) the system can be used for children under 2 years of age, and b) it may be able to distinguish more general UNDD children from TD children.”

Please see P. 51 of DSM-5 on assigning DSM-IV diagnoses using DSM-5 format.

Thanks for the point. The phrase is modified, which is now in lines 476-480, as follows:

“The trained classifier was also tested on 57 participants between 10 to 18 months of age. The classifier screened two boys from the rest, i.e. Child1 and Child2 (Table 3). Child1 showed evidences of genetic disease and diagnosed with developmental delay and Child2 received UNDD classification by our experts. This suggests that a) the system can be used for children under 2 years of age, and b) it may be able to distinguish other neurodevelopmental disorders.”

NOTE: It should be mentioned that the number of this group of participants is the same as before (57) but one boy’s parents did not cooperate during the follow-up and he was removed from the participants. Instead, another boy who was 11 months old and was detected as ASD using our classifier was added to participants. He is indicated in Table 3 as Child1.

The Discussion section is the first time this 2nd dataset is mentioned (no mention in the methods, results, etc.). Please provide adequate details on this analysis in earlier sections as relevant.

Thanks for this clarifying point. We have added the details of those participants at the end of the Participants section (lines 165-176). A table is added which consists of the information about the participants of this set who were diagnosed with disorders or there were evidences of developmental problem or other clinical problems after following up at age 3 or more. In fact this part is added to the ms as all those participants are now older than 3 years. We followed up them by asking questions which were provided by experts to evaluate if they are in their normal developmental stage.

“Although this approach was trained and tested on children older than 18 months, we tested the approach on 57 participants between 10 to 18 months to investigate how it works on children under 18 months. These 57 participants consisted of 28 boys and 29 girls with the mean of 15.2 for both and standard deviations of 2.8 and 2.9 respectively. All these participants were evaluated afterward at the age of 3 or more by the same follow-up procedure using our expert-selected questionnaire.. At the time of initial voice collection, 55 of these participants had no evident or diagnosed disorder. Two of them were referred to our experts due to the positive results of screening by our method. The diagnosis or concerns for the two mentioned participants as well as the participants with any evidence of having abnormality in the developmental milestones during the follow-up procedure are summarized in Table 3. The summary of disorders is given in the last column of Table 3 is based on the parental interviews and our experts’ evaluation. Unfortunately, Child5, Child6, and Child7’s parents did not cooperate to get experts’ evaluation.”

Appendix.
This section contains key methods information necessary to understand analytical approach and is short enough to be in the main body of the ms. The ms is currently missing these details. Please move this information into the main body under the Methods section.

We added a subsection to the method section, namely “Details of the implementation” and moved this section there.

P. 23, 24, 25
Please address the following queries related to preprocessing of the data:
A1) Are these data continuous? i.e. have you retained original continuity of the data (i.e. as the data were acquired?). If not, how instances were strung together needs to be explained.

Yes each selected segments consists of a continuous part of the signal. We believe that this is implicitly included the in ms, in the following sentence in lines 204-5.

“Each remaining continuous segment of the cries was considered and used as a sample (instance) in this study”

A2) Pipeline/preprocessing:
A 2a) How are technical issues of data preprocessing addressed here? (how are known problems addressed?)

The data processing has been addressed in more details in the revised version. We hope that this addresses the reviewer’s concern. In the discussion section, we have added the following sentence to further address this concern:

“Finally, automating the preprocessing part is a technical issue that should be handled if the cry voice-based screening is planned to be fully automated. This would be important since such a screening system can be deployed in systems such as Amazon Alexa [58] to automatically screen problematic cry voices.”

A 2b) illustration of raw data of interest (cry instances of ASD and TD participants)

We can have two image of cry voices of the two groups. However, since there is no visual difference between the two, it may not help readers.

A 2c) equal length of samples/instances? All of this information is missing

This has been addressed in the comments above and in lines 205-207 of the ms as follows:

“Finally, since the basic voice features were extracted from 20 milliseconds frames, to generate statistical features of the basic features, the minimum length of the cry segments were set to 15 frames, i.e. 300 milliseconds.”

A3a) What software/scripts was used to handle the raw data (.wav files)? (What is the software used for input/output? Open source?)

To cut and select the segments of the signals Sound Forge Pro 11.0 was used.
A3b) What software was used to program the classifiers (MATLAB, Python, etc.; which toolboxes or libraries were used if any?)

The classifiers was implemented in Python using scikit-learn library. This has been added in the subsection titled “Details of the implementation” in line 307.

A4) Appendix – P. 24
“This feature supports our expert’s report saying variations in the cry of ASD children is more than TD children.”
A4a) Please present this report/ anecdotal variations in cry sooner in the ms.

The report is now indicated in the Introduction section as follows:

“It should be mentioned that the final selected features, in this study, are common among our set of children with ASD between 18 to 53 months of age. These selected features support experiential knowledge of our experts stating that the variations in the cry of children with ASD are more than TD children.”

A4b) This information touching upon variability parameters in ASD cry data is highly significant and should be elaborated upon in the Discussion. There are now many papers in the ASD field reporting that autism biological signals (from children and adults with ASD and work with infants at high risk for autism) are characterized by increased/heightened levels of variability (e.g. Denisova & Zhao, 2017).

It is now added to discussion section as follows in lines 462-6:

“On the other hand, the heightened variability in the two features, found in this study, for children with ASD is significant due to the reports from other studies [17, 55] which shows increased biological signals variability in children with ASD and infants at high risk for autism in comparison to TD children. These features are statistical features of the cry instances that hold constant, at least, across an age range studied in this research.”

Your result is in line with these other reports from many groups worldwide and uniquely adds to the growing research indicating that a marker of higher variability is an important feature in ASD. Perhaps that is why you find it age-invariant: it is not cry per se, but statistical features of the cry instances that hold constant across age. Please be sure to discuss this point in the Discussion.

6. PLOS authors have the option to publish the peer review history of their article ([what does this mean?](https://journals.plos.org/plosone/s/editorial-and-peer-review-process#loc-peer-review-history)). If published, this will include your full peer review and any attached files.


If you choose “no”, your identity will remain anonymous but your review may still be made public.


**Do you want your identity to be public for this peer review?** For information about this choice, including consent withdrawal, please see our [Privacy Policy](https://www.plos.org/privacy-policy).

Reviewer #1: No

Reviewer #2: No

Reviewer #3: No


[NOTE: If reviewer comments were submitted as an attachment file, they will be attached to this email and accessible via the submission site. Please log into your account, locate the manuscript record, and check for the action link "View Attachments". If this link does not appear, there are no attachment files to be viewed.]


While revising your submission, please upload your figure files to the Preflight Analysis and Conversion Engine (PACE) digital diagnostic tool, <https://pacev2.apexcovantage.com/>. PACE helps ensure that figures meet PLOS requirements. To use PACE, you must first register as a user. Registration is free. Then, login and navigate to the UPLOAD tab, where you will find detailed instructions on how to use the tool. If you encounter any issues or have any questions when using PACE, please email us at [figures@plos.org](mailto:figures@plos.org). Please note that Supporting Information files do not need this step.

*In compliance with data protection regulations, you may request that we remove your personal registration details at any time.* [*(Remove my information/details)*](https://www.editorialmanager.com/pone/login.asp?a=r)*. Please contact the publication office if you have any questions.*
