# Supplementary material for: Early screening of autism spectrum disorder using cry features
Source: PLoS One. 2020 Dec 10;15(12):e0241690. doi: 10.1371/journal.pone.0241690 (PMC7728261; doi:10.1371/journal.pone.0241690)
Supplement: Attachment — Submitted filename: Response to reviewers.docx [file pone.0241690.s002.docx]

Reviewer's Responses to Questions

**Comments to the Author**

1. If the authors have adequately addressed your comments raised in a previous round of review and you feel that this manuscript is now acceptable for publication, you may indicate that here to bypass the “Comments to the Author” section, enter your conflict of interest statement in the “Confidential to Editor” section, and submit your "Accept" recommendation.

Reviewer #1: All comments have been addressed

Reviewer #3: (No Response)

2. Is the manuscript technically sound, and do the data support the conclusions?

The manuscript must describe a technically sound piece of scientific research with data that supports the conclusions. Experiments must have been conducted rigorously, with appropriate controls, replication, and sample sizes. The conclusions must be drawn appropriately based on the data presented.

Reviewer #1: Yes

Reviewer #3: Yes

3. Has the statistical analysis been performed appropriately and rigorously?

Reviewer #1: Yes

Reviewer #3: Yes

4. Have the authors made all data underlying the findings in their manuscript fully available?

The [PLOS Data policy](http://www.plosone.org/static/policies.action#sharing) requires authors to make all data underlying the findings described in their manuscript fully available without restriction, with rare exception (please refer to the Data Availability Statement in the manuscript PDF file). The data should be provided as part of the manuscript or its supporting information, or deposited to a public repository. For example, in addition to summary statistics, the data points behind means, medians and variance measures should be available. If there are restrictions on publicly sharing data—e.g. participant privacy or use of data from a third party—those must be specified.

Reviewer #1: Yes

Reviewer #3: (No Response)

5. Is the manuscript presented in an intelligible fashion and written in standard English?

PLOS ONE does not copyedit accepted manuscripts, so the language in submitted articles must be clear, correct, and unambiguous. Any typographical or grammatical errors should be corrected at revision, so please note any specific errors here.

Reviewer #1: No

Reviewer #3: No

6. Review Comments to the Author

Please use the space provided to explain your answers to the questions above. You may also include additional comments for the author, including concerns about dual publication, research ethics, or publication ethics. (Please upload your review as an attachment if it exceeds 20,000 characters)

Reviewer #1: The authors have been very responsive to the last round of reviews and I'm happy with the manuscript as is; no suggested edits on my part

Reviewer #3: I thank the authors for carefully addressing previous points made. I am marking this as a major revision because I would like to see the authors carefully and thoroughly address the following new and remaining points.

1) The abstract, the final version of which was not available in the previous submission, requires substantial changes and must be fully re-written to include rationale for the current work. More details are needed about the analytic methods (including acquisition methods) and conclusion. First of all, the implications/conclusion of the work is completely missing and must be added. Overall, it should reflect all of the changes and edits in the current ms.

To address the reviewer’s concerns, the abstract has changed as follows:

“The increase in the number of children with autism and the importance of early autism intervention has prompted researchers to perform automatic and early autism screening. Consequently, in the present paper, a cry-based screening approach for children with Autism Spectrum Disorder (ASD) is introduced which would provide both early and automatic screening. During the study, we realized that ASD specific features are not necessarily observable in all children with ASD and in all instances collected from each child. Therefore, we proposed a new classification approach to be able to determine such features and their corresponding instances. To test the proposed approach a set of data relating to children between 18 to 53 months which had been recorded using high-quality voice recording devices and typical smartphones at various locations such as homes and daycares was studied. Then, after preprocessing, the approach was used to train a classifier, using data for 10 boys with ASD and 10 TD boys. The trained classifier was tested on the data of 14 boys and 7 girls with ASD and 14 TD boys and 7 TD girls. The sensitivity, specificity, and precision of the proposed approach for boys were 85.71%, 100%, and 92.85%, respectively. These measures were 71.42%, 100%, and 85.71% for girls, respectively. It was shown that the proposed approach outperforms the common classification methods. Furthermore, it demonstrated better results than the studies which used voice features for screening ASD. To pilot the practicality of the proposed approach for early autism screening, the trained classifier was tested on 57 participants between 10 to 18 months. These 57 participants consisted of 28 boys and 29 girls and the results were very encouraging for the use of the approach in early ASD screening.”

2) “Due to the importance of automatic and early autism screening” Please consider providing additional rationale for the importance of early ASD screening. It is mentioned in passing in the ms on P. 1 but I would like to see some more elaboration for why it is important to detect ASD (or ASD signs) in the main body of the ms /in the Introduction.

What we have written for the importance of early screening was folllow:

“Considering the fact that early diagnosis is crucial for effective treatments [7, 9]”

Now it is changed to:

“It is of the utmost importance to have early diagnosis/screening in order to provide early intervention which is more effective at the first few years of life than later on [7, 9-11]. It is shown that early intervention improves the developmental performance in children with ASD [12]. It has also been reported that early interventions would be cost saving for families and the treatment service systems [13, 14]. Consequently, there are two main questions: 1) can autism be screened earlier than 18 months to reduce the typical diagnosis or intervention age and 2) is it possible to employ intelligent methods for the screening of autism to eliminate the widespread need for experts?”

3) There remain many instances of awkward language use, which often leads to confusion about the intended meaning. I would like to see a version of this ms after appropriate language edits have been implemented, because I do not think that the Authors have adequately dealt with the language issues in this version. I would suggest the use of a professional editing service, or have a colleague who is a native British English or American English speaker help edit the ms.

It used editing service by NativeEnglishEdit.com for this time and the certification letter is provided as a supplementary file.

4) Some examples of awkward language use, but there are others. Also please be very clear about the intended meaning. There is confusion about screening for ASD manifestations in children below 18 months *who have not yet been diagnosed with an ASD* and confirming ASD in children below 18 mo – please clarify what is meant by using clear language.

We should apologize for the confusion and fixed it as follows:

In line 51, we added the following sentence to clearly show our intention:

“It should be mentioned that our goal was to answer these questions with respect to screening all children who may not have clear symptoms. The screened children should go through a diagnosis procedure to acquire confirmation and/or be cautiously worked with.”

In line 55, we updated the sentence replacing screening with diagnosis. “Fortunately, there are studies in the literature showing that the age of diagnosis can be lower than 18 months.“ we changed screening to diagnosis to correctly refer to the intended action by the referenced studies and methods.

“53 As mentioned above, there are studies tried to screen children with ASD under 18 months,”
Should read: “As mentioned above, multiple studies attempted to screen children for ASD below 18 months of age”

“widespread expertness for autism diagnosis“
Should read: “expertise for diagnosing autism”

Thanks for the correction. It is changed now in line 49.


5) Some references are missing in the ms – For example, it does not seem that this reference is in the main text.
Paliwal KK, Lyons JG, Wójcicki KK, editors. Preference for 20-40 ms window duration in speech analysis. 2010 4th International Conference on Signal Processing and Communication Systems; 2010: IEEE.

In the previous version of the manuscript, we only provided this reference in the response letter for the reviewer, assuming that it is not necessary for readers in general. Nonetheless, based on this feedback, we included the reference in the manuscript as follows:

“Finally, since the basic voice features were extracted from 20 milliseconds frames [50], to generate statistical features of the basic features, the minimum length of the cry segments were set to 15 frames, i.e. 300 milliseconds.”

6) The fact that ADOS is not available at all in Farsi is still not mentioned in the body of the ms. I think it is important to mention this fact, in case some readers wonder why you did not administer the ADOS to your participants. Please address in the main body of the ms why participants were not administered the ADOS.

It is now added in line as follows:

“It should be noted that ADOS, which is a very common diagnostic tool is not administered widely in Iran since there is no official translation of ADOS in Farsi. “

7. PLOS authors have the option to publish the peer review history of their article ([what does this mean?](https://journals.plos.org/plosone/s/editorial-and-peer-review-process#loc-peer-review-history)). If published, this will include your full peer review and any attached files.


If you choose “no”, your identity will remain anonymous but your review may still be made public.


**Do you want your identity to be public for this peer review?** For information about this choice, including consent withdrawal, please see our [Privacy Policy](https://www.plos.org/privacy-policy).

Reviewer #1: No

Reviewer #3: No

[NOTE: If reviewer comments were submitted as an attachment file, they will be attached to this email and accessible via the submission site. Please log into your account, locate the manuscript record, and check for the action link "View Attachments". If this link does not appear, there are no attachment files.]

While revising your submission, please upload your figure files to the Preflight Analysis and Conversion Engine (PACE) digital diagnostic tool, <https://pacev2.apexcovantage.com/>. PACE helps ensure that figures meet PLOS requirements. To use PACE, you must first register as a user. Registration is free. Then, login and navigate to the UPLOAD tab, where you will find detailed instructions on how to use the tool. If you encounter any issues or have any questions when using PACE, please email PLOS at [figures@plos.org](mailto:figures@plos.org). Please note that Supporting Information files do not need this step.
